# Juvenile Dermatomyositis: Updates in Pathogenesis and Biomarkers, Current Treatment, and Emerging Targeted Therapies

**DOI:** 10.1007/s40272-024-00658-2

**Published:** 2024-10-19

**Authors:** Hanna Kim

**Affiliations:** National Institute of Arthritis Musculoskeletal and Skin Diseases, National Institutes of Health, Bethesda, MD USA

## Abstract

Juvenile dermatomyositis is a rare systemic inflammatory autoimmune disease involving muscle, skin, and vessels. Most patients do not fully respond to initial therapy, instead having a chronic refractory or polycyclic disease course. Pathogenesis is not completely understood, but immune cell dysregulation, particularly of B cells, mitochondrial dysfunction, changes in neutrophils and neutrophil extracellular traps (NETs), and increased type I and type II interferon (IFN) signaling have been described. There are limited randomized controlled trials of drugs in juvenile dermatomyositis (JDM), and treatment is largely based on lower-quality data such as case series, retrospective studies, and open-label prospective studies. These data have been compiled into expert recommendations or consensus treatment plans, which help guide therapy. While initial therapy is more standard with most including corticosteroids (high-dose oral and/or pulse intravenous methylprednisolone) and methotrexate, for refractory patients, guidelines are more varied with multiple options or combinations, including biologic therapies. There is a clear need for more efficacious and personalized therapy in JDM. Emerging treatment options worthy of further study in JDM include targeting IFN-signaling (JAK, IFNAR1, IFN beta), B-cells (CD20, CD19, BAFF, TACI, CD38, BCMA) including Chimeric Antigen Receptor (CAR)-T cell therapy, mitochondrial dysfunction, and NETs.

## Key Points

**Table:** 

There are no approved therapies for juvenile dermatomyositis (JDM). Consensus treatment plans or recommendations guide treatment primarily based on case series, retrospective studies, and uncontrolled open-label studies.
Most patients have refractory disease indicating better treatments are needed.
Better understanding of pathogenesis and biomarkers will help identify more targeted therapies that may be more efficacious.
There are drugs targeting pathogenic IFN-signaling, B-cells, mitochondrial dysfunction and neutrophil extracellular traps (NETs) that show promise in JDM, but further study is needed.

## Introduction

Juvenile dermatomyositis (JDM) is the most common idiopathic inflammatory myopathy (IIM) of childhood, although still rare with an overall incidence of about 3 per million in the USA [1]. It commonly causes pathology in skin, muscle, and vessels with other systemic findings. Etiology is not fully understood. Myositis-specific autoantibody (MSA) groups define clinical subgroups with associations to specific clinical features, disease course, and sometimes treatment response within the more heterogeneous umbrella of JDM. For example, anti-TIF1 (p155/140 or TIF1-gamma) is overall the most common (18–35% in Japan, USA, and UK) associated with photosensitivity, cutaneous ulceration, and chronic or polycyclic disease [2–4]. Anti-NXP2 (MJ) is overall the next most common (15–26% in Japan, USA, and UK) associated with myalgia, dysphonia, dysphagia, calcinosis, more severe weakness, and less remission after 2 years [2–6]. Anti-MDA5 (CADM-140) is more common in Japan (32%) than in the USA and UK (6–8%), associated with less muscle weakness, ulceration, arthralgia, and arthritis, as well as interstitial lung disease and systemic features like fever and weight loss [2–4, 7, 8]. There are also other less-common MSA groups in JDM reviewed elsewhere in detail [9–11].

Although mortality has significant decreased after corticosteroids were introduced as a standard therapy, mortality remains about 1–5% [12–14]. Up to three-quarters of patients with JDM have a chronic or polycyclic disease course [13, 15], some with disability or disease-related damage [13, 16]. There remains much room for improvement in management. This review will highlight pathogenesis and biomarkers as context to identify better treatments, as well as an overview of current and evolving treatments.

## Pathogenesis and Biomarkers

Broadly, the pathogenesis of JDM is not fully understood. At a basic level, it is thought to be a combination of environmental triggers such as ultraviolet (UV) exposure and infections, and genetic risk factors [9, 10, 17]. More recent studies have focused on inflammatory and immune dysregulation as pathomechanisms. Clinically, muscle enzymes are standardly used to help monitor for muscle inflammation or muscle disease activity, but they are not always elevated with clear disease activity [18–20]. Thus, there is a need for better disease biomarkers, which may also help us better understand pathomechanisms. Better understanding pathogenesis and biomarker identification may help us repurpose existing treatments or develop novel treatments based on new treatment targets.

### Immune Cell Subsets

Inflammatory immune cell infiltrate including T and B cells has been observed in JDM muscle biopsies [21]. This has been further characterized as part of muscle biopsy scoring [22]. Peripheral immune subsets and related gene expression were evaluated in multiple studies. B cells and genes related to B-cell survival such as *BAFF* were found to correlate with disease activity in adult IIM and/or JDM [23, 24]. Plasmablasts and plasma cells are B cells that secrete antibodies and 60–70% of patients with JDM have myositis-specific autoantibodies, which are theorized to be part of disease pathogenesis [9–11]. Immature transitional B cells were found to be expanded prior to treatment in JDM peripheral blood, also correlating with disease activity [25, 26]. There was also an increase in naïve B cells and CD4^+^ T cells noted peripherally in JDM [25, 27, 28]. Additionally, peripheral T regulatory cells were found to be in the same proportion in active JDM as in controls, but functionally impaired [29]. CD16^+^ monocytes and natural killer (NK) cells were found to be decreased in the blood of patients with JDM at baseline or prior to treatment [25, 27, 28, 30].

### Mitochondrial Biomarkers

Broad gene expression data from JDM muscle identified mitochondrial dysfunction from functional enrichment analysis [31]. Peripheral CD14^+^ monocytes were noted to have decreased mitochondrial-associated gene expression in JDM versus controls [32]. Mitochondrial dysfunction has also been identified by increased circulating mitochondrial DNA and MT-ND6 (mitochondrial NADH dehydrogenase 6) protein in JDM versus healthy controls. Additionally, anti-mitochondrial autoantibodies were found to correlate with creatine kinase (CK) and calcinosis in patients with JDM [33]. JDM mitochondria were also noted to have increased oxidative stress or reactive oxygen species (ROS) with increased oxidized mitochondrial DNA intracellularly. This (oxidized mitochondrial DNA) was found to also stimulate the innate immune system with increased interferon-stimulated gene expression (*MX1*) [32].

### Neutrophils and Neutrophil Extracellular Traps (NETs)

As an overview of these features in autoimmune disease, neutrophils are an innate immune cell that function as a first-line cell-mediated defense with killing of microorganisms. Increasing literature points to their importance in autoimmune diseases including lupus and arthritis. Neutrophils are typically purified using a density gradient, with neutrophils expected to separate with the dense mononuclear cells. However, some low-density granulocytes (LDGs), which do not separate with the dense mononuclear cells, have been identified in multiple inflammatory conditions including cancer, autoinflammation, and autoimmunity. These LDGs have with a different phenotype and function including an increased tendency to form neutrophil extracellular traps (NETs). NETs are networks of extracellular DNA and chromatin with granules, mitochondrial DNA, and histones. NETs are extruded in response to danger signals such as cytokine activation or reactive oxygen species to entrap and kill fungi, bacteria, and viruses. In autoimmune diseases, if there is decreased NET degradation or increased NET formation (NETosis), there may be (1) increased immune activation and inflammation, which can cause damage, and (2) increased autoantigen exposure, both of which continue the cycle of immune activation and inflammation recruiting the adaptive immune system [34, 35].

Regarding these features in myositis as potential biomarkers, increased LDGs was associated with weakness in JDM as well as activity in skin and vasculopathy, while increased NET markers were associated with muscle and lung disease activity [36]. NETs were observed in JDM muscle containing mitochondrial DNA. There was increased neutrophil activation noted by calprotectin and peroxidase activity in JDM plasma [37]. In adult DM muscle, an increased neutrophil gene signature was found that correlated with an increased IFN signature [36]. This was supported by increased neutrophil degranulation and NET functional themes in JDM and adult DM gene and protein expression [38].

### Endothelial/Vascular Biomarkers

Vasculopathy is thought to be an important part of disease pathogenesis based on vascular abnormalities noted on pathology as well as nailfold and gingival capillary changes observed on physical examination [10, 11, 39, 40]. Additionally, a vascular remodeling theme has been identified in JDM muscle transcriptomic data [41]. In adult DM muscle, dysregulated angiogenesis themes from gene and protein expression have been identified [42].

Multiple angiogenesis and endothelial activation markers correlate with disease measures or clinical features in JDM. Increased circulating endothelial cells (CECs) and microparticles, which are markers of vascular damage, have been reported and generally correlate with disease activity. CECs were also noted to correlate with abnormal nailfold capillaries and galectin-9 (Gal-9), an IFN-related protein marker mainly made by endothelial cells [43, 44]. In serum, markers of endothelial activation (vascular cell adhesion protein 1 or VCAM-1) and inhibition of angiogenesis (angiopoietin-2 and soluble vascular endothelial growth factor receptor 1 or VEGFR1) correlated with disease activity [45]. Expression of VCAM-1 was increased and microRNA or miRNA-126, a VCAM-1 inhibitor, was decreased in muscle biopsies from untreated JDM versus pediatric controls. Additionally, peripheral VCAM-1 and endoglin correlated with clinical vasculopathy by nailfold capillary end-row loop scoring [46].

### Interferon (IFN)

Interferon (IFN) and interferon-related gene and protein expression have been consistently shown to be increased in key tissues (blood, muscle, and skin) from patients with JDM [47]. IFN alpha was found to be increased in JDM peripheral blood versus controls by a single-molecule array assay called Simoa [48]. Increased IFN-regulated gene and interferon-regulated protein expression has been found consistently in blood by multiple studies, also correlating with disease activity [47, 49–51]. Of note, in France, a six-gene nanostring IFN-related gene score is included as part of routine assessment [52]. Regarding IFN-related proteins, multiple have been identified that correlate with disease activity [40, 53]. Of note, two of these proteins, galectin-9 and CXCL10 (C-X-C motif chemokine ligand 10 or interferon gamma-induced protein 10/ IP-10), were validated as disease activity-related biomarkers in multiple separate international patient populations with examples of elevation prior to clinical flares in JDM indicating potential to identify flares prior to clinical-onset [54]. Assessed with flow cytometry, sialic acid binding Ig-like lectin 1 (SIGLEC-1) on monocytes is another IFN-related protein marker that correlates significantly with disease activity in JDM [55, 56].

In JDM muscle biopsies, increased type I (IFN alpha, IFN beta) and type II (IFN gamma) interferon-regulated gene scores have been demonstrated, with increased IFN gamma also shown by immunohistochemistry. These type I and type II IFN scores correlated with histopathology findings such as T-cell infiltration and perifascicular atrophy [57]. In adult DM muscle, increased interferon-regulated gene expression was specifically found in areas of perifascicular atrophy [42]. In JDM muscle, higher type II IFN-regulated gene scores also correlated with a longer time to clinically inactive disease [57]. Other type I IFN-related markers (MxA protein or myxovirus resistance protein A, *ISG15* gene or interferon-stimulated gene 15) in muscle were found to correlate with muscle disease activity [58, 59]. An increased type I and type II IFN-related gene expression has also been shown in adult DM and JDM skin [60, 61].

Interestingly, when IFN alpha is added to skeletal muscle cells in-vitro, this also increases mitochondrial reactive oxygen species (ROS) [33]. In vitro, when IFN alpha is added to  human dermal microvascular endothelial cells (HMEC-1), there is decreased angiogenesis which is rescued with ruxolitinib, a janus kinase inhibitor [62]. Also, in Sect. 2.2 above, oxidized mitochondrial DNA, increased in JDM, was found to increase interferon-stimulated gene expression.

IFN also was tied to other biomarkers above. In Sect. 2.3, a neutrophil gene signature was associated with an IFN gene signature in adult DM muscle. In Sect. 2.4, abnormal nailfold capillaries (vasculopathy) were associated with higher IFN-related markers such as galectin-9 in JDM. Thus, IFN is tied to multiple biomarker or mechanism in JDM/DM including mitochondrial dysfunction, neutrophils/NETs, and vascular changes.

## Current Treatment Recommendations (Consensus Treatment Plans or Guidelines)

As the exact cause of JDM is not fully understood, treatments are generally less specific, targeting immune cell activation and inflammation (Table 1) [9, 11, 17, 39, 63]. Many treatments were originally used as chemotherapy, with lower dosing for JDM. There are no drugs approved specifically for JDM, at least in the USA. While there are many important parts of treatment that are not drugs, such as exercise and sun protection, they are beyond the scope of this review.

**Table 1:** Juvenile dermatomyositis therapeutics mechanisms of action for current therapies

Therapeutic	Mechanism of action
Corticosteroids^a^	Multiple including decreased PMNL migration and decreased cytokine production
Methotrexate	Block de novo purine synthesis
Mycophenolate mofetil	Block de novo purine synthesis
Cyclosporine	Calcineurin inhibition, IL-2-dependent T-cell activation
Tacrolimus	Calcineurin inhibition, IL-2-dependent T-cell activation
Hydroxychloroquine	Exact mechanisms unknown—antigen-antibody interactions, chemotaxis
Azathioprine	Block de novo purine synthesis
Intravenous immunoglobulin	Exact mechanism unknown—cytokine suppression, inhibit immune cell activation
Rituximab	Chimeric monoclonal Ab to CD20 (deplete B cells)
Infliximab	Chimeric monoclonal Ab to TNF alpha
Adalimumab	Human monoclonal Ab to TNF alpha
Abatacept	Human CTLA4 and Fc of IgG blocking costimulatory CD28 on T cells
Tocilizumab	Human monoclonal Ab to IL-6
Cyclophosphamide	Alkylating agent that interferes with DNA replication
Plasma exchange	Remove antibodies, complement, immune complexes

*Ab* antibody, *CTLA4* cytotoxic T-lymphocyte associated protein 4, *Fc* fragment crystallizable region, *IgG* immunoglobulin G, *IL* interleukin, *PMNL* polymorphonuclear leukocyte, *TNF* tumor necrosis factor

^a^Includes prednisone and methylprednisolone and equivalents

Large randomized interventional drug studies are quite limited (*n* = 2) in this rare disease, which limits the high-quality data needed to justify a specific drug indication for JDM. The Rituximab In Myositis (RIM) randomized double-blind placebo-phase clinical trial with 48 refractory patients with JDM and 152 with refractory adult myositis published in 2013 did not meet its primary end point, but did find benefit from rituximab in multiple validated measures, particularly with the JDM cohort [64]. The Paediatric Rheumatology International Trials Organisation (PRINTO) network completed a multicenter, randomized, open-label study comparing three treatments (prednisone alone, prednisone with methotrexate, prednisone with cyclosporin) in new-onset JDM, finding more met the definition of improvement when taking prednisone with a steroid-sparing agent, and methotrexate had less side effects than cyclosporin [65].

As an alternative, there are management guidelines generally informed by literature review, consensus, and/or expert opinion. In North America, there are consensus treatment plans or CTPs led by the Childhood Arthritis and Rheumatology Research Alliance (CARRA). The CARRA CTPs are based on surveys to assess current practice and expert opinion to develop consolidated treatment protocols for further study. Multiple countries or regions have guidelines or consensus statements for treatment. Overall, most of the medications included in these recommendations or CTPs (Table 1) are based on lower levels of evidence (case reports, case series, cohort studies) as reviewed in detail elsewhere [9, 11, 17, 39]. These medications are generally immunosuppressive or immunomodulatory and an overview of their mechanisms is included in Table 1 [9, 11, 17, 39, 63].

### Initial Treatment

There are CARRA CTPs based on surveys for current practice as well as expert opinion to achieve consensus treatment regimens for further study regarding for initial management of moderate JDM and skin predominant JDM [66–68]. For moderate JDM, all plans start with methotrexate (MTX) and high-dose prednisone, with an option to add pulse dose intravenous (IV) methylprednisolone (IVMP), or both IVMP and IV immunoglobulin (IVIG). For skin predominant JDM, all plans include hydroxychloroquine (HCQ) with an option to add MTX or both MTX and high-dose prednisone (Table 2A) [66–68].

**Table 2:** Medical therapy recommendations or consensus treatment plans for (A) initial and (B) refractory JDM

A. Initial therapy										
JDM Subgroup	Organization	Reference	High dose prednisone	MTX^a^	IVMP	IVIG	MMF	CsA	AZA	CYC
Moderate JDM	CARRA	Huber et al. 2010 [67]	×	×	Op	Op				
Skin predominant	CARRA	Kim et al. 2017 [68]	Op	Op						
JDM, not severe^b^	SHARE	Belutti Enders et al. 2017 [69]	×	×	×					
JDM, severe^b^	SHARE	Belutti Enders et al. 2017 [69]	×	×	×					A/Sw op
Moderate/severe JDM	GKJR	Hinze et al. 2018 [70]	×	×	×	Op	MTX alt	MTX alt	MTX alt	
JADM	SRGPRD	Kobayashi et al. 2020 [71]								
Mild/moderate JDM	SRGPRD	Kobayashi et al. 2020 [71]	× or IVMP	×	× or GC				MTX alt	
Severe JDM	SRGPRD	Kobayashi et al. 2020 [71]			×				Op	×
JDM with ILD	SRGPRD	Kobayashi et al. 2020 [71]			×			CNI and/or CYC		CYC and/or CNI
Muscle disease	BSR	Oldroyd et al. 2022 [72]	× or IVMP	×	× or GC	Cons (sev)	Alt (+ skin)			Cons (sev)

B. Refractory JDM																
JDM subgroup	Organization	Reference	High dose prednisone	MTX^a^	IVMP	IVIG	MMF	CsA	AZA	RIT	TNFi (ADA, INF)	ABAT	TOCI	CYC	Biologic agent	PE
Moderate JDM	CARRA	Huber et al. 2012 [67]			Op	Op	Op	Op	Op						Op (unspecified)	
Persistent skin	CARRA	Huber et al. 2017 [73]				Op	Op	Op								
Biologic DMARD	CARRA	Tarvin et al. 2024 [74]	Op		Op	Op				Arm	Arm	Arm	Arm			
JDM, not severe	SHARE	Belutti Enders et al. 2017 [69]				Op	Sw op	Sw op		Sw op	Sw op					
JDM severe	SHARE	Belutti Enders et al. 2017 [69]				Op		Op		Op	Sw op					
Moderate/severe JDM	GKJR	Hinze et al. 2018 [70]				A/Sw op	A/Sw op	A/Sw op	A/Sw op	A/Sw op	A/Sw op^c^			A/Sw op		
JADM	SRGPRD	Kobayashi et al. 2020 [71]	Op and/or MTX	Op and/or GC												
Mild/moderate JDM	SRGPRD	Kobayashi et al. 2020 [71]				X	Cons	A/Sw CNI op		Cons				A/Sw op		Cons
Severe JDM	SRGPRD	Kobayashi et al. 2020 [71]				A/Sw CNI op	Cons	A/Sw CNI op		Cons				A/Sw op		Cons
JDM with ILD	SRGPRD	Kobayashi et al. 2020 [71]				A/Sw CNI op	Cons	A/Sw CNI op		Cons				A/Sw op		Cons
Muscle Disease	BSR	Oldroyd et al. 2022 [72]				Cons				Cons				Cons		
Skin disease	BSR	Oldroyd et al. 2022 [72]				Cons				Cons						

*A/Sw op* add-on or switch option, *Alt* alternative, *Arm* one of four biologic options, *Cons* consider, *Op* add-on option, *Sw op* option to switch, *ABAT* abatacept, *ADA* adalimumab, *AZA* azathioprine, *BSR* British Society of Rheumatology, *CARRA* Childhood Arthritis and Rheumatology Research Alliance, *CNI* calcineurin inhibitor, *CsA* cyclosporin, *CYC* cyclophosphamide, *DMARD *disease-modifying antirheumatic drug, *GC* high dose oral glucocorticoid, *GKJR* Society for Pediatric Rheumatology in Germany and Austria, *HCQ* hydroxychloroquine, *ILD* interstitial lung disease, *INF* infliximab, *IVIG* intravenous immunoglobulin, *IVMP* intravenous methylprednisolone, *JADM* juvenile amyopathic dermatomyositis, *JDM* juvenile dermatomyositis, *Mod* moderate, *MMF* mycophenolate mofetil, *MTX* methotrexate, *PE* plasma exchange, *RIT* rituximab, *S or Sev* severe, *SRGPRD* Scientific Research Group for Pediatric Rheumatic Diseases supported by the Japanese Ministry of Health, Labor, and Welfare, *SHARE* Single Hub and Access point for pediatric Rheumatology in Europe, *Tacro* tacrolimus, *TNFi* tumor necrosis factor alpha inhibitor, *TOCI* tocilizumab

×: part of published treatment recommendation or consensus treatment plan

^a^All specified preference for subcutaneous methotrexate other than BSR and SRGPRD

^b^SHARE recommendations for initial management were also specified for flare

^c^Tumor necrosis factor alpha inhibitor not specified

Single Hub and Access point for pediatric Rheumatology in Europe (SHARE) guidelines have been developed for JDM [69]. These are part of a European initiative to optimize and disseminate diagnostic regimens in Europe, providing guidelines beyond just medical therapy. Regarding therapeutics for initial disease and flare, high-dose corticosteroids, preferably IVMP pulse doses followed by high dose oral steroids, are recommended with MTX. For initial disease or flare if severe, the same options are recommended with consideration for adding or switching to cyclophosphamide (CYC) (Table 2A) [69].

The Society for Pediatric Rheumatology (GKJR) in Germany and Austria developed consensus-based statements regarding the diagnosis, treatment, and monitoring of moderate and severe JDM [70]. Regarding medical treatment, initial options are glucocorticoid treatment with oral and intravenous corticosteroid regimens described. Initial therapy recommendations include MTX as a disease-modifying anti-rheumatic drug (DMARD), with azathioprine (AZA), mycophenolate mofetil (MMF), and cyclosporine (CsA) as alternatives to MTX in the case of intolerance (Table 2A) [70].

The Scientific Research Group for Pediatric Rheumatic Diseases (SRGPRD) supported by the Japanese Ministry of Health, Labor, and Welfare published Clinical Practice Guidelines for JDM, including specifics for therapeutic management [71]. For mild or moderate JDM, high-dose oral prednisolone or IVMP (pulse) is recommended with MTX, with AZA as an alternative for MTX. For severe JDM, IVMP with CYC and optional AZA is recommended. For JDM with ILD, IVMP with a calcineurin inhibitor (CNI) such as CsA or tacrolimus and/or CYC is recommended (Table 2A) [71].

The British Society for Rheumatology (BSR) Standards Audit and Guidelines Working group created evidence-based guidelines for idiopathic inflammatory myopathy spanning juvenile and adult-onset disease for management, including specific medical therapeutic options [72]. Specific to JDM, they specify guidelines for how to treat skeletal muscle inflammation (myositis) and noted this should be managed with oral or IV corticosteroids and MTX. They noted that IVIG and CYC should be considered for severe disease, also considering MMF as an alternative to MTX for those with muscle and skin disease (Table 2A) [72].

### Refractory Disease Treatment

As mentioned, despite having multiple treatment options and treatment recommendations available, most patients have chronic or polycyclic disease. Here we will review available guidelines and CARRA CTPs for refractory disease, including combination treatment (Table 1). This need for refractory disease guidelines emphasizes the need for more efficacious treatment options and personalized treatment options [9–11, 17, 39].

For the CARRA moderate JDM CTP with refractory disease, it is recommended to consider adding IVMP pulse, IVIG, CsA, MMF, AZA, and/or an unspecified biologic agent [67]. For the CARRA skin resistant CTP, plans options are IVIG MMF, or CsA [73]. Regarding the biologic DMARDs in refractory moderate JDM CTP, there are four arms or choices including TNF-alpha inhibitor or TNFi (adalimumab or infliximab), abatacept, rituximab (RIT), and tocilizumab (Table 2B) [74]. Of note, abatacept was recently studied in an open-label pilot study in refractory JDM and nine of ten patients met the definition of improvement at 24 weeks [75]. The development of these CTPs began before JAK inhibitors were being frequently considered in refractory JDM, so they were not included [74].

From SHARE guidelines for refractory JDM without severe disease, treatment intensification by adding IVIG or switching to MMF, CsA, RIT, or TNFi (adalimumab or infliximab) is recommended. For refractory severe disease, adding RIT, changing to TNFi, or adding CsA and IVIG is recommended (Table 2B) [69].

From GKJR Society of Rheumatology guidelines in Germany and Austria, if refractory, options are to add or switch to IVIG, MMF, CsA, AZA, RIT, TNFi (unspecified), or CYC (Table 2B) [70]. From the SRGPRD in Japan, for amyopathic JDM, adding systemic glucocorticoids and/or MTX was recommended. For refractory mild or moderate JDM, IVIG is recommended, and then adding or switching to CNI or CYC with consideration of MMF, RIT and plasma exchange. For refractory severe JDM or refractory JDM with ILD, adding or switching to IVIG, CNI, or CYC is recommended with consideration of MMF, RIT, and plasma exchange (Table 2B) [71]. From the British Society of Rheumatology (BSR), consideration of IVIG, RIT and CYC for juvenile refractory myositis (muscle inflammation). For juvenile refractory skin disease, they recommend consideration of IVIG and RIT (Table 2B) [72].

In summary, all initial treatment recommendations or CARRA CTPs have high dose corticosteroids as a mainstay of initial treatment for moderate to severe JDM. Other than for skin predominant (CARRA CTP) and severe JDM and JDM with ILD (SRGPRD), all also recommend MTX. Most also recommend IVMP at least as an option. Some recommendations include MTX alternatives (GKJR, SRGPRD). For severe disease, CYC is often named as a treatment or option (SRGPRD, SHARE, BSR). For refractory disease, there is more variation in recommendations from different parts of the world, though IVIG is the most common. Considerations include MMF, CsA, AZA, CYC, and biologic agents including RIT, TNFi, abatacept, and tocilizumab, as well as plasma exchange.

## Emerging Treatments

Given the prevalence of refractory patients with JDM or patients with a chronic or polycyclic disease course, identifying additional treatment options that may have higher efficacy and/or decreased side effects is of great importance. Here, targeted treatments based on pathogenic mechanisms and biomarkers with reports in JDM, adult myositis are highlighted when available. Otherwise, targeted treatments are highlighted from similar conditions such as lupus, which also has autoantibodies and dysregulated B cells [76–78], increased interferon signaling [77–79], NETosis [34, 35, 77, 78], and mitochondrial dysfunction [77, 78, 80], as they indicate potential for broader use in JDM (Table 3).

**Table 3:** Juvenile dermatomyositis therapeutics mechanisms of action for emerging therapies

Therapeutic	Mechanism of action
Janus kinase inhibitors	Inhibit JAK signaling, including IFN signaling
Ocrelizumab	Humanized monoclonal Ab to CD20 (deplete B cells)
Ofatumumab	Human monoclonal Ab to CD20 (deplete B cells)
Obinutuzumab	Humanized monoclonal Ab to CD20 (deplete B cells)
Obexelimab	Bifunctional chimeric monoclonal Ab to CD19 and FcGammaRIIb to downregulate B cells
Belimumab	Human monoclonal Ab to BAFF to inhibit B cell survival, differentiation
Blisibimod	Human fusion protein to BAFF to inhibit B cell survival, differentiation
Atacicept	Humanized fusion protein to TACI (receptor of BAFF, APRIL) to inhibit B-cell survival, differentiation
Telitacicept	Humanized fusion protein to TACI (receptor of BAFF, APRIL) to inhibit B-cell survival, differentiation
Daratumumab	Human monoclonal Ab to CD38 for plasma cell depletion
CD19 CAR T cells	Engineered chimeric antigen receptor to CD19 on T-cell immunotherapy to kill cells with CD19
BCMA CAR T cells	Engineered chimeric antigen receptor to BCMA on T-cell immunotherapy to kill cells with BCMA
*N*-acetylcysteine	Antioxidant, reactive oxygen species scavenger
Metformin	Inhibit mitochondrial complex 1
Anifrolumab	Humanized monoclonal Ab to IFNAR1 (type I IFN receptor)
Dazukibart	Humanized monoclonal Ab to IFN beta

*Ab* antibody, *BAFF* b-cell activating factor, *BCMA* b-cell maturation antigen, *CAR* chimeric antigen receptor, *FcGammaRIIb* fragment crystallizable fragment of gamma receptor IIb, *IFN* interferon, *IFNAR1* interferon alpha and beta receptor subunit 1, *JAK* janus kinase, *TACI* transmembrane activator and CAML interactor

### Therapies Targeting IFN signaling

IFN has consistently been reported as an important pathway in JDM with IFN-related markers increased in a disease activity-related manner. Additionally, IFN seems to also be related to mitochondrial dysfunction, NETs, and vascular changes making it a particularly intriguing treatment target.

#### Janus Kinase Inhibitors

IFN signals through janus kinases (JAKs) and Signal Transducers and Activators of Transcription (STATs). One way to target IFN signaling is by using JAK inhibitors or jakinibs, though JAK inhibitors inhibitor multiple cell-signaling molecules, not just IFN [81–84]. JAK inhibitors were also identified as a potentially efficacious treatment for inflammatory myopathies from a high-throughput screen of over four thousand compounds assessing a skeletal muscle model with type I IFN exposure inducing major histocompatibility complex 1 (MHC1) as a reporter [85].

The first report of JAK inhibitor use in JDM was with ruxolitinib in 2018 [86]. Since then, there have been more than 100 patients with JDM reported on jakinibs (ruxolitinib, tofacitinib, baricitinib) from primarily case reports, case series, and retrospective studies [81, 82, 87]. There have been two small open prospective studies [88, 89] and no controlled studies. Most patients had active skin disease (98%) while only about a third (34%) had muscle disease. The vast majority of patients with active skin and/or muscle disease had improvement with JAK inhibitors, including by photography and magnetic resonance imaging (MRI) (objective measures), though controlled quality data is lacking. There are limited reports of improvement of interstitial lung disease particularly with anti-MDA5 autoantibody, and stabilization or improvement of calcinosis. The vast majority of jakinib reports are in refractory patients with JDM (98%) and in many cases, the improvement with jakinib was noted to be impressive given lack of improvement with multiple other medications [81–84, 87]. With growing literature on jakinibs in JDM and the impressive nature of improvement in some cases, awareness of this option for treatment in the JDM community is growing rapidly.

There are rare cases reported when a jakinib is used as part of initial therapy when severe, and currently there is an open-label study of baricitinib (MYOCIT) as part of first-line therapy in Paris, France (NCT05524311). There are varied dosing regimens that have been reported including higher dosing as has been used for Mendelian interferonopathies [81, 88, 90–92]. Multiple studies noted a decrease of IFN-related markers on jakinib therapy indicating that the increased efficacy of jakinibs may be attributed to their ability to more effective decrease IFN signaling versus other treatment options, but larger controlled studies are needed [81, 82, 87]. Regarding safety, herpes simplex and varicella zoster infections have been reported with jakinibs including in JDM. BK viral titers should be monitored as they have been noted to be increased. Changes in cell counts have been noted as with jakinib use in other conditions. Elevation of different muscle enzymes have been reported in JDM studies without muscle or strength changes, similar to elevation of liver enzymes and creatine kinase (CK) noted in rheumatoid arthritis studies with jakinibs. Thus, changes in muscle enzymes in JDM when on a jakinib should be interpreted with caution and clinical context, as they may be an adverse effect of the jakinib [81, 82, 87, 93].

#### Other IFN Blockade

Another way to block IFN-signaling is to block the receptor of type I IFN (IFN alpha, IFN beta), IFNAR1. Anifrolumab is a humanized monoclonal antibody to IFNAR1 which has been approved for treatment of systemic lupus erythematosus (SLE). In a small cohort of pediatric autoinflammatory interferonopathies (*n* = 12) who had persistently elevated interferon-regulated gene scores on high-dose baricitinib, anifrolumab was given. The patients did not have any disease flares and normalized interferon-regulated gene scores. Most were also able to wean baricitinib and steroids [94]. An adult with anti-TIF1 dermatomyositis associated with metastatic cancer was noted to have improvement of muscle disease on corticosteroids and IVIG, but persistent skin disease. Anifrolumab 300 mg monthly IV was given and marked skin improvement was noted after a single dose [95]. More recently, anifrolumab was also reported with a 14-year-old girl with anti-TIF1 JDM with refractory skin disease. She has previously been treated with corticosteroids, tofacitinib up to 20 mg/day (high dose), and seven other immunomodulatory treatments with improvement of muscle disease, but persistent skin disease and persistently elevated interferon beta. Anifrolumab (300 mg every 4 weeks) was started with improvement of rash within 72 h of the first infusion documented by skin rash photos and at decreased disease activity by validated JDM skin assessments at 2 months [96].  This may be a potentially efficacious option even if refractory to JAK inhibitor treatment.

Dazukibart is a humanized monoclonal antibody to IFN beta, previously known as PF-06823859. This has been studied in a phase 2 randomized controlled study (NCT03181893) of refractory adult DM. A total of 57 skin-predominant adult patients with DM were noted to have decreased itch by week 1 and decreased skin disease activity at week 4. Eighteen muscle-predominant adult patients with DM were noted to have decreased CK at 4 weeks and decreased patient-reported disease activity at 4 weeks, with increased overall improvement at week 12. Overall, the most common adverse event was noted to be gastrointestinal, without herpes zoster or herpes simplex reported [97, 98]. Thus, there are multiple current options for targeting IFN that seem to be clinically promising for JDM.

### Therapies Targeting B-cell Signaling

Given the increased B-cells and autoantibody production in JDM, B cells in general are a reasonable treatment target with multiple specific potential targets (Tables 3, 4) [99–102]. Rituximab, which targets CD20 with a chimeric monoclonal antibody, has had proven benefits as mentioned above by randomized controlled trial [64]. However, 50% of myositis patients took longer than 20 weeks to respond with rituximab [64] and given that immunogenicity or intolerance that can develop with rituximab, a chimeric product, as well as non-response (lack of efficacy) [64, 99, 100, 103], other options could include human or humanized monoclonal autoantibodies to CD20. Ocrelizumab, ofatumumab, and obinutuzumab are human or humanized monoclonal antibodies to CD20 that also lead to B-cell depletion. Ocrelizumab was studied in a phase III study of lupus nephritis and improvement was noted, though they did not meet the primary outcome [104]. Two adults with anti-SRP immune-mediated necrotizing myopathy, for which rituximab is commonly used, were treated with ofatumumab. Ofatumumab was chosen due to concern for rituximab intolerance or adverse effects and both patients had notable improvement [105, 106]. There was an anti-Jo1 adult myositis patient had response to obinutuzumab after lack of response to rituximab, indicating these options should be considered in JDM for those patients refractory to rituximab [107].

**Table 4:** B-cell marker expression during development and differentiation, and drugs associated

Drugs by B-cell marker	Marker	Pro-B cell	Pre-B cell	Transitional B cell	Naïve B cell	Germinal center B-Cell	Memory B cell	Plasmablast	Plasma cell
Obexelimab, CD19 CAR T-cells	CD19	×	×	×	×	×	×	×	+/−
Rituximab, ocrelizumab, ofatumumab, obinutuzumab	CD20		×	×	×	×	×	+/−	
Daratumumab	CD38	×	×	×		×		×	×
Atacicept, telitacicept	TACI						+/−	×	+/−
BCMA CAR T-cells	BCMA							×	×

*CAR* chimeric antigen receptor, *TACI* transmembrane activator and CAML interactor, *BCMA* B-cell maturation antigen

Regarding other B-cell markers, obexelimab is a bifunctional chimeric monoclonal antibody that targets CD19 and the Fc (fragment crystallizable) gamma receptor IIb to downregulate B cells. This was evaluated in a phase II adult SLE study and noted improvement in patients, though they did not meet their primary outcome. BAFF, a circulating B-cell survival marker, is targeted by belimumab as a human monoclonal antibody to inhibit B-cell survival and differentiation. This was studied in a randomized double-blind, placebo-controlled study in refractory adult myositis and found to have benefit, though it did not meet its primary endpoint [108]. Another drug targeting BAFF via a human fusion protein, blisibimod, was studied in a phase 3 adult SLE randomized controlled trial and demonstrated improvement, but did not meet the primary outcome [109]. There were also some studies with drugs that target TACI (transmembrane activator and CAML interactor), the receptor for BAFF and APRIL (a proliferation-inducing ligand) as a humanized fusion protein (atacicept, telitacicept). Atacicept was studied in a phase 2b trial of adult SLE with improvement noted, though they did not meet the primary outcome [110]. Telitacicept was studied in a phase 2b trial of active adult SLE and met the primary outcome, leading to its approval for SLE in China [111]. Thus, there are multiple other targets for B cells that warrant further study in JDM.

Regarding plasma cells, there is a human monoclonal antibody to CD38 which depletes plasma cells called daratumumab. Three case reports with adult myositis have noted benefit with IV daratumumab. All patients had clinically significant disease affecting swallow and/or breathing. One patient with anti-SRP immune-mediated necrotizing myopathy had dysphagia and significant weakness affecting respiration requiring intubation. Two individuals with anti-MDA5 had rapidly progressive interstitial lung disease, one intubated and one on high-flow nasal cannula. All had persistently positive autoantibodies despite rituximab treatment and refractory active disease despite three to six other agents. IV daratumumab was administered at 5–8 months with notable clinical improvement by one month and decreased or undetectable autoantibody levels [112–114].

B-cell targeting therapies beyond rituximab appear to provide efficacy potentially even if rituximab was not effective, in some cases with a decreased adverse effect profile. Daratumumab, targeting plasma cells, seems to be an option to consider when acutely ill and rapid efficacy is critical (e.g., respiratory compromise).

### Autologous Chimeric Antigen Receptor (CAR) T-Cell Therapy

#### Overview of CAR T-Cell Therapy

An emerging therapy of particular interest in autoimmune disease is autologous CAR T-cell therapy to B-cell targets [115, 116]. Briefly, the concept of CAR T-cell therapy is to target things in the body to be eliminated with the precision and responsiveness of an antibody and the potent cytotoxicity, mobility, and ability to expand and contract of a T cell. A CAR T-cell is engineered combining the antibody-derived variable regions (VH, VL) to allow for design of a target, with T-cell receptor-derived constant regions so that this engineered T-cell can maintain recognition as self (one’s own immune system rather than foreign). This has been optimized to increase potency and persistence including the addition of co-stimulatory domains. With autologous CAR T-cell therapy, T-cells are isolated from blood withdrawn (leukapheresis). T-cells are then genetically altered and engineered to target a specific antigen/cell and then generated for re-infusion. This autologous CAR T-cell single infusion is a “living drug” unique to an individual and expensive, requiring significant technology and manufacturing expertise to produce [115, 116], with other methods of CAR T-cell therapy (allogeneic) continuing to evolve [117],

In oncology, CAR T-cell therapy has been a remarkably successful immunotherapy for multiple cancers even when other therapies have failed by combining efficient killing/depletion of target cells or markers, with specificity to limit off-target killing and side-effects. Of note, high-levels of target cell killing over a short period of time with CAR T-cell therapy can result in cytokine release syndrome (CRS), an adverse event clinicians outside of oncology are generally less familiar with, which can present with fever and/or organ-specific or multi-organ failure [115, 118].

Despite caveats, the treatment success including for B-cell malignancies has raised the possibility of using CAR T-cell therapy in autoimmune disease. This could potentially reset the B-cells in the immune system with a single infusion to manage disease without needing to continue potent combination immunosuppression typically used in severe or refractory autoimmune disease, which carry significant side effects [115, 116]. Additionally, in SLE, existing B-cell targeting such as rituximab has been found to not always fully eliminate B cells including memory B cells, and B cells in lymph nodes and tissues, which may limit its efficacy [119–123]. CAR T-cells on the other hand can expand and survive wherever they are exposed to their target antigen with better tissue penetration, which has shown to more completely deplete B-cells when B-cells are targeted [124].

#### CAR T-Cell Therapy in Myositis

B-cell targeting by autologous CAR-T cell therapy in adults with autoimmune disease has included five with myositis as well as most recently one with juvenile dermatomyositis with different B cell targets (Table 4) [99–101]. Of the five adults, four patients had antisynthetase syndrome of one to five-year duration, all with muscle disease and interstitial lung disease and two with skin disease. They were treated with CD19 CAR T-cell therapy [125–128]. One patient with anti-SRP immune-mediated necrotizing myopathy with significant weakness of 7-year duration was treated with BCMA or B-cell maturation antigen CAR T-cell therapy [129]. All five were refractory, previously treated with RIT and three to seven other medications with 5–18 months of follow-up after CAR T-cell therapy. All had significant clinical improvement. After B-cell reconstitution, four remained off all medications with one only on MMF. Thus, refractory patients were able to discontinue all medications after a single CAR T-cell infusion for four cases, and only remain on one medication in the fifth case. Regarding safety, all developed cytokine release syndrome (CRS), though four of five were grade 1, the mildest form, and one had grade 2 CRS. One developed immune-effected cell-associated neurotoxicity grade 1, the mildest form [125–129].

One patient with JDM (10 years old), who appears to be the first juvenile autoimmune disease patient to receive CAR T-cell therapy, was reported to receive CD19 CAR T-cell therapy. He was antinuclear antibody (ANA) positive with no myositis-specific or myositis-associated autoantibodies. He had chronic muscle and skin disease activity with ulcerations and calcinosis without notable lung, gastrointestinal, or cardiac involvement. He had been on prednisone, MTX, CsA, MMF, and HCQ. After transferring to the current treating center, IVIG and CYC was started. Later, he was given plasmapheresis and RIT and seemed to have some improvement attributed to RIT. Four months later, he had some worsening and was re-dosed with RIT. He had another flare 4 months later and was given CYC and plasmapheresis without much improvement. At that point, CD19 CAR T-cell therapy was given. All other immunomodulatory treatment was stopped per protocol for the leukapheresis, which was associated with some mild worsening of disease. After his CD19 CAR T-cell infusion, his circulating B cells became undetectable, as did B cells on a bone marrow aspirate at 2 and 4 weeks post-CART-cell infusion. Compared with the time of CAR T-cell infusion (baseline), at 2 weeks, he had stable skin disease and mild cytokine release syndrome associated with myalgia limiting muscle assessment. However, at 4 weeks, there was significant improvement in muscle and skin disease by validated measures, as well as notable improvement in calcinosis, with resolution of ulcers. B cell recovery occurred at 8 weeks with continued clinical improvement by validated strength and skin disease activity measures, also with normalization of MRI and interferon scores by week 24. At 8 months post CAR T-cell therapy (last follow-up reported), the patient continued to do well off of all immunosuppression [130]. Thus, CAR T-cell therapy targeting B cells seems to have powerful potential to achieve disease control in JDM without need for continued medication, though further study is needed.

### Therapies Targeting Mitochondrial and Neutrophil Extracellular Traps (NETs)

As JDM is refractory for many patients and seems to have multiple facets to its pathogenesis including mitochondrial dysfunction and NETs, we should explore atypical therapies in JDM that target these pathogenic mechanisms. *N*-acetylcysteine (NAC) is an antioxidant and reactive oxygen species scavenger with potential to address mitochondrial dysfunction. This has been studied in an experimental autoimmune myositis (EAM) murine model and was noted to prevent mitochondrial dysfunction, muscle weakness, as well as prevent an increase in interferon-stimulated gene score [131]. In peripheral blood mononuclear cells from patients with JDM prior to treatment, NAC was noted to decrease interferon-stimulated gene expression [32]. In a randomized controlled trial of NAC in adult SLE, NAC 1800 mg/day was noted to decrease validated SLE disease activity scores at 3 months [132].

Mitochondrial DNA has been noted in neutrophil extracellular traps or NETs [35]. Outside of its indication in diabetes, metformin is an antioxidant that can inhibit mitochondrial complex 1. In vitro, when healthy control neutrophils are treated with metformin, there is decreased NET formation and decreased mitochondrial DNA in NETs. When metformin was added as a therapy for mild to moderate SLE in a randomized controlled open-label study versus conventional therapy, those with metformin had decreased flares over 12 months indicating benefit [133]. Thus, NAC and metformin may merit further study in JDM for added benefit.

## Conclusion

JDM is a complex disease with multiple contributors to pathogenesis. While current therapies have dramatically decreased mortality, most patients still do not respond to initial therapy and have a refractory chronic or polycyclic disease course. Current consensus treatment plans or guidelines lead to improvement for most, but better options are needed for refractory patients as well as better predictors for who will respond to which treatment (personalized medicine). Targeting pathogenic pathways will improve our ability to manage the disease with more success (higher efficacy).

The rapidly growing literature of refractory JDM improving on janus kinase inhibitors points to the potential added efficacy based on targeting IFN signaling. This particular efficacy with targeting of IFN may also correlate with how IFN is related to other pathology observed in JDM, mitochondrial dysfunction, NETs, and vascular changes. There is less data about anti-IFNAR1 and anti-IFN beta but there are promising limited reports with rapid response in adult DM and patients with JDM. There are multiple B-cell targeting therapies that are promising, including human or humanized monoclonal antibodies to CD-20 and treatments for other B cell targets (BAFF, TACI, CD19). Notable and rapid benefit was noted with plasma-cell depletion via anti-CD38 daratumumab which can be considered particularly for critically ill patients. CAR T-cell therapy targeting B cells (CD19 or BCMA) has great potential to clinically improve and possibly reset the B-cell immune system and subsequently allow discontinuation of all immunomodulatory medication, with limited reports including in JDM. NAC and metformin are interesting options that also may improve treatment efficacy targeting mitochondrial dysfunction and NETs in JDM. Further study on biomarkers to predict specific drug response and larger controlled studies of targeted therapies will help us optimize treatment for patients with JDM.
